# Is Anodal Transcranial Direct Current Stimulation an Effective Ergogenic Technology in Lower Extremity Sensorimotor Control for Healthy Population? A Narrative Review

**DOI:** 10.3390/brainsci12070912

**Published:** 2022-07-13

**Authors:** Changxiao Yu, Songlin Xiao, Baofeng Wang, Jiaxin Luo, Cuixian Liu, Junhong Zhou, Weijie Fu, Jing Jin

**Affiliations:** 1 Key Laboratory of Exercise and Health Sciences of Ministry of Education, Shanghai University of Sport, Shanghai 200438, China; yuchangxiao1014@163.com (C.Y.); xiao_songlin@126.com (S.X.); wangbaofeng1911@163.com (B.W.); l1060822405@163.com (J.L.); liucuixian@sus.edu.cn (C.L.); 2 The Hinda and Arthur Marcus Institute for Aging Research, Hebrew SeniorLife, Harvard Medical School, Boston, MA 02131, USA; 3 Shanghai Frontiers Science Research Base of Exercise and Metabolic Health, Shanghai 200433, China; 4 School of Psychology, Shanghai University of Sport, Shanghai 200438, China

**Keywords:** neural activity, standing postural control, gait, time-on-task, cognitive tasks

## Abstract

Anodal transcranial direct current stimulation (a-tDCS) aims to hone motor skills and improve the quality of life. However, the non-repeatability of experimental results and the inconsistency of research conclusions have become a common phenomenon, which may be due to the imprecision of the experimental protocol, great variability of the participant characteristics within the group, and the irregularities of quantitative indicators. The aim of this study systematically summarised and analysed the effect of a-tDCS on lower extremity sensorimotor control under different experimental conditions. This narrative review was performed following the PRISMA guidelines until June 2022 in Web of Science, PubMed, Science Direct, Google Scholar, and Scopus. The findings of the present study demonstrated that a-tDCS can effectively improve the capabilities of lower extremity sensorimotor control, particularly in gait speed and time-on-task. Thus, a-tDCS can be used as an effective ergogenic technology to facilitate physical performance. In-depth and rigorous experimental protocol with larger sample sizes and combining brain imaging technology to explore the mechanism have a profound impact on the development of tDCS.

## 1. Introduction

Sensorimotor control in the lower extremity (i.e., balance, gait, mobility, and postural control) is the fundamental element for everyday activities, often coinciding with non-postural cognitive tasks [1,2]. Such adaptive sensorimotor processes are exceedingly complex, which integrate the somatosensory, visual, and vestibular transmission pathways [3]. In addition to integrating external information, the activation of the brain regions is also key for maintaining the execution of neural signals and strategies targeting cortical excitability that will help the sensorimotor control in the lower extremity [4,5].

**Figure 1:**
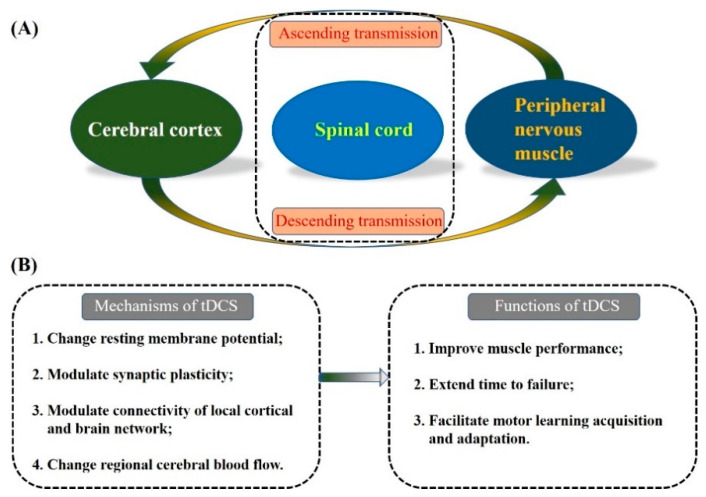
Information pathways between the cortex and the musculoskeletal system (**A**), and the mechanisms and functions of tDCS (**B**).

Non-invasive brain stimulation techniques such as therapeutic tools have been developed to treat neuropsychiatric and neurological disorders [6]. Transcranial direct current stimulation (tDCS) is one of such techniques that modulates the excitabilities of brain regions by sending low-intensity current to polarise and depolarise the resting membrane potentials of neurons via surface scalp electrodes [7]. The mechanisms and functions of tDCS are shown in Figure 1. As a neuromodulation technique, anodal-tDCS (a-tDCS) has positive effects on enhancing synaptic connections [8] and modulating the nervous system, thereby improving the coordination efficiency of musculoskeletal modification during the performance of lower extremity sensorimotor control [9], such as balance and gait in healthy adults. Specifically, early studies have provided insights into the potential ergogenic effect of a-tDCS on a wide range of exercise types based on promising outcomes [7,10,11]. For instance, multi-session a-tDCS can improve semantic associations for schizophrenia patients, which supports its neuromodulation role in improving cognitive functions [12]. By investigating the changes of a-tDCS on cortical plasticity, Pisoni et al. [13] elucidated the positive correlation between the neurophysiological effects of a-tDCS at specific cortical circuits and cognitive enhancement.

Still, the effect of a-tDCS reported in previous studies was inconsistent, which may be due to the selection of tDCS variables (i.e., duration, montage, location, etc.) being of high variance. For instance, applying 20-min a-tDCS over the cerebellum for young adults may increase the excitability of the motoneuron pool, which can result in a continuous neural drive for the motor neurons and improve the dynamic balance task while standing with two feet on a movable platform [14]. However, the enhancements have not been found in another study [2], showing that the missing ergogenic effects of a-tDCS may be the shorter duration (10 min vs. 20 min) and the small sample size. Additionally, in the dual-tasking condition (i.e., standing or walking while performing another task), brain regions are involved in cognitive processes [15,16]. Studies also demonstrated that a-tDCS contributes to modulating cortical excitability and results in a sustained neural drive for the motor neurons, which may have enabled better integration amongst different sets of nuclei necessary for the execution of cognitive-motor tasks [17]. Therefore, more brain regions of interest have been included as stimulation targets (i.e., primary motor cortex (M1), prefrontal cortex (PFC), supplementary motor area (SMA), and temporal cortex (TC)).

Therefore, this narrative review aimed to systematically characterise the effect of a-tDCS on the lower extremity sensorimotor control in healthy individuals, providing constructive knowledge on the optimal protocol design and effects of a-tDCS on lower extremity sensorimotor control to inform future studies.

## 2. Materials and Methods

### 2.1. Search Strategy

This narrative review was performed for relevant papers from the first data available until June 2022 in the following databases: Web of Science, PubMed, Science Direct, Google Scholar, and Scopus. The following key search terms were used to improve the matching of the searched English literature with the research purpose: ‘transcranial direct current stimulation’ or ‘tDCS’ or ‘HD-tDCS’ and ‘postural control’ or ‘balance’ or ‘sensorimotor control’ or ‘physical performance’ or ‘gait’ or ‘time-on-task’. Moreover, the reference lists of the included studies were reviewed to find additional relevant studies that have not appeared in the database with our initial electronic search terms.

### 2.2. Eligibility Criteria

Studies that met the following requirements were included: (a) English full-text articles; (b) randomised, single/double-blinded, sham-controlled experimental design; (c) the intervention of a-tDCS was performed in healthy adults; (d) application of bilateral a-tDCS or unilateral a-tDCS in any brain region; (e) perform lower extremity sensorimotor testing with static or/and dynamic postural control. In addition, review, conference, and unpublished articles were excluded.

### 2.3. Overview of the Included Studies

**Figure 2:**
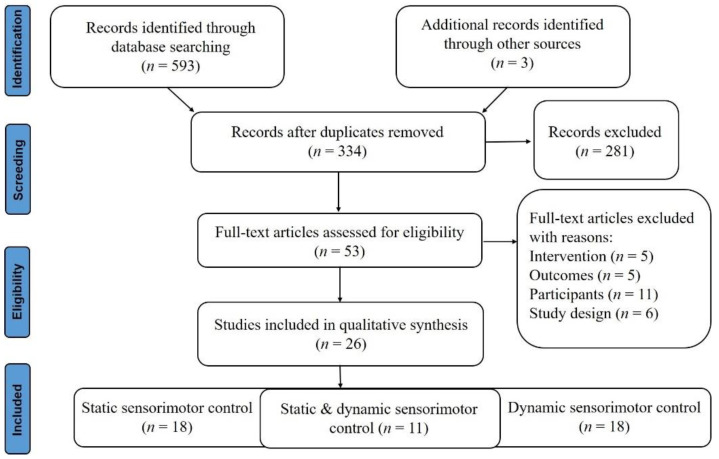
PRISMA summary of the study selection process.

We collected a total of 587 relevant documents from the Web of Science, PubMed, Science Direct, Google Scholar, and Scopus. After rigorous screening, 26 studies were used in the narrative review (static sensorimotor control, 18; dynamic sensorimotor control, 18; static and dynamic sensorimotor control, 11) (Figure 2). As shown in Table 1, only one study simultaneously recruited two populations (young and older adults) as participants [14] among all included studies, and three studies compared and investigated two sets of montage placement [9,14,18]. In addition, participants in 13 studies (56.5%) received two simulation methods (a-tDCS and sham stimulation) separately, with an interval of 3 to 7 days or more, and 8 of these studies (61.5%) selected 7 days. Only two studies (8.7%) applied a multi-session stimulation program [18,19]. Nine studies (39.1%) used electrode sponges of different sizes for the cathode and anode, and only one study used high-definition tDCS (HD-tDCS) [20].

**Figure 3:**
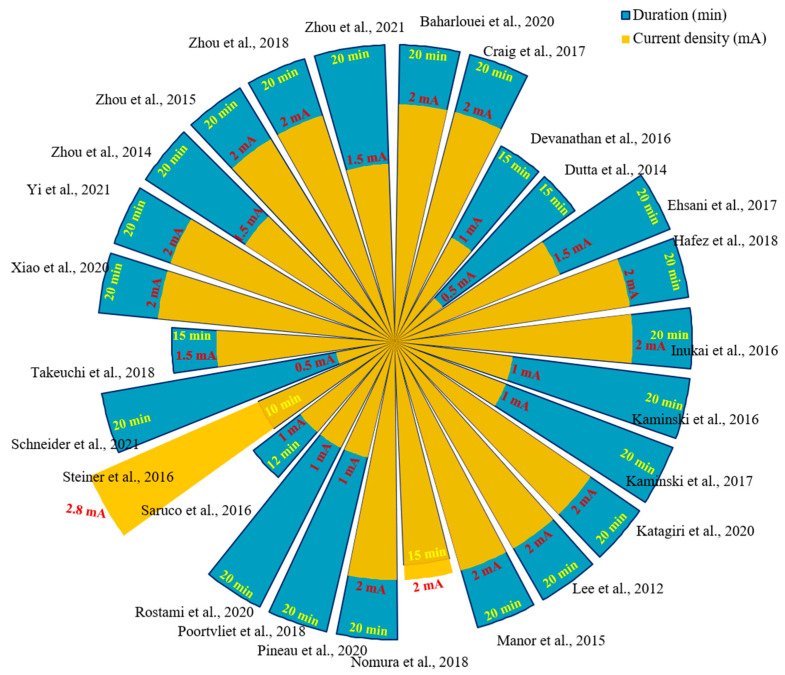
Stimulation duration and intensity in included studies [2,9,11,14,18,19,20,21,22,23,24,25,26,27,28,29,30,31,32,33,34,35,36,37,38,39].

All included studies applied a randomised design, of which two studies (7.7%) applied a parallel design, and others (92.3%) applied a crossover trial design. Studies assessed a total of 680 participants, with a population number of 25.81 ± 12.06 (mean ± SD) per study (from 5 to 57). In addition, the studies of Ehsani et al. [11] and Hafez et al. [18] had a total of 5 (14.7%) and 4 (10.3%) dropouts, respectively. Regarding gender, all the studies included 292 male participants and 359 female participants, one of which did not indicate gender [21]. Three studies (11.5%) only recruited male participants, and no study only recruited female participants. Across the studies, 269 participants (40.09%) were under the age of 50, and 402 (59.91%) were over 50 years old. As shown in Figure 3, concerning the stimulus duration of a-tDCS, the majority of studies (76.92%) used 20 min. The current density was primarily 2 mA, with mean ± SD current density per the study of 1.61 ± 0.57 mA (ranging from 0.5 mA to 2.8 mA) and electrode size of 26.69 ± 13.67 cm^2^ (from 1 cm^2^ to 55.25 cm^2^). The anode montage was placed in the motor cortex (51.61%), cerebellum area (25.81%), PFC (19.35%), and TC (3.23%).

## 3. Practical Considerations

### 3.1. Effect of A-tDCS on Standing Postural Control

Standing upright is a complex task, which occurs simultaneously with non-postural cognitive tasks. Such ‘dual tasking’ significantly increases the difficulty of lower extremity sensorimotor control compared with ‘single tasking’, and it is often used as an important evaluation index for the elderly to prevent falls. Older adults with executive dysfunction are linked to poor dual-tasking capacity, leading to greater risk of falls [40]. Relevant literature indicates the effectiveness of a-tDCS of the dorsolateral prefrontal cortex (dlPFC) on performing two cognitive tasks concurrently. Manor et al. [41] reported that as compared to sham, 20 min of a-tDCS induced significant improvements in dual-task postural sway speed and areas in older adults with functional limitations, but not in single-task standing postural control performance. In addition, they argued that the reduced dual-task costs were due to tDCS improving the capacity of the frontal-executive systems and optimising cognitive-motor resources. In line with the studies on young healthy adults, Zhou et al. [37] also found that the dlPFC was a primary brain region supporting cognitive dual tasks. However, one study partially replicated the study of Zhou et al. [37], and the results were inconsistent. Pineau et al. [31] investigated the postural performance in a simple and dual-task with eyes open and closed via assessing the centre of pressure (COP) parameters immediately after a 20 min a-tDCS session. The results showed that acute a-tDCS cannot effectively improve dual-task performance, and they explained that the discrepancy may be due to the physical activity level of participants. Moreover, the application of slightly larger current intensity (2 mA vs. 1.5 mA) and smaller stimulating electrodes (25 cm^2^ vs. 35 cm^2^) for the latter may not have a decisive effect on the experimental results compared with the former. Based on the feature of ceiling effects, the more energetic the participants are, the more difficult it is to reflect the positive effectiveness of a-tDCS. A better understanding of the effect of a-tDCS on standing posture control can be established by investigating the age-related loss of complexity in healthy older adults. Therefore, Zhou et al. [38] quantified the complexity of postural sway of the elderly with a-tDCS over the left PFC in single and dual-task postural control using multi-scale entropy. Their results indicated that a-tDCS was associated with an increase in prefrontal cortical excitability, which coincided with improved complexity of standing postural sway specifically within a dual-task condition.

The effects of a-tDCS on other cortical regions have also been investigated. The cerebellum is a pivotal stimulus target, and as a complex intracranial organ, it has an extensive connection with many areas of the midbrain, brainstem, and cerebral cortex [42]. A large number of studies have confirmed that cerebellar a-tDCS can enhance the links and increase the control function of the cerebellum on the motor cortex, vestibular system, and other brain regions [11,43,44]. Standing postural control includes both static control, that maintains stability on a firm and unchanging support surface, and dynamic control, that maintains balance on a movable platform. Ehsani et al. [11] investigated the effect of cerebellar a-tDCS on static and dynamic postural control in older individuals using a Biodex Balance System, and they revealed that the participants receiving cerebellar a-tDCS showed significantly reduced postural sway in anterior–posterior and medial–lateral directions. Similarly, combined with postural control training, cerebellar a-tDCS stimulation can improve the skill acquisition of postural control in young individuals [27]. As we previously mentioned, tDCS is a form of neuromodulation, which can modulate neural activity. Therefore, a-tDCS of the M1 has gained increasing interest as a neurorehabilitation tool for facilitating the excitability of this region and enhancing standing performance. Xiao et al. [20] reported that the static standing balance performance with eyes closed improved after single-session HD-tDCS by assessing the averaged sway velocity of the centre of gravity in anterior–posterior and medial–lateral directions. However, no significant differences were observed between HD-tDCS and sham stimulation amongst young participants. These results were attributed to the small sample size and ceiling effect. In addition, the results were in agreement with the study of Inukai et al. [24], confirming that a-tDCS over the cerebellum cannot enhance the standing posture control capacity of young health populations compared with sham stimulation. Literature indicates that the equivocal results in standing posture control are due to the stimulation target and age.

Two studies were included for comparison of M1 a-tDCS to determine the effect of cerebellar a-tDCS on standing posture control [9,18]. In the lower extremity sensorimotor control of healthy individuals, a previous study has asserted that the motor cortex plays a smaller role compared with the cerebellum and subcortical structures [45]. Moreover, a recent study has shown that the cerebellum and M1 a-tDCS have significant effects on the standing posture balance of the elderly [9]. However, Hafez et al. [18] found that posture training combined with bilateral cerebellar or M1 a-tDCS was more effective than cerebellar a-tDCS alone or postural training alone in improving the anterior–posterior and medial–lateral stability index of standing postural control under eyes open and closed conditions. An important aspect of the divergence between the two studies was the difference in the experimental protocol.

Although the aforementioned findings were combined, the systematic information reconfirmed that a-tDCS over the dlPFC could improve standing posture control performance under dual-task conditions, particularly for the elderly. In any case, further optimisation of experimental protocol could provide a stable experimental effect on standing posture control. Therefore, some speculation on the a-tDCS mechanism should be treated with caution.

### 3.2. Effect of A-tDCS on Gait Speed and Time-on-Task

The improvements in gait speed after a 20 min session of a-tDCS over the prefrontal cortex under single-task conditions were found compared with sham stimulation, but the differences were not statistically significant [29,37]. Although the aforementioned result is encouraging, evidence shows that most studies have a small sample size. Regarding the gait speed in double-task conditions, four studies proved that a-tDCS over the prefrontal cortex can significantly reduce dual-task costs by assessing the walking tests in healthy elderly and young adults [29,34,37,39]. Another study with the same test protocol did not find a significant functional improvement in walking with dual tasking based on the TUG test of mobility in functionally limited older adults [41]. Factors such as the dose and duration of a-tDCS in participants with different physical conditions should be appropriately adjusted. Previous studies have indicated that anticipatory postural adjustments were generated from the increased excitability of the SMA to promote gait posture stability for healthy adults. Collectively, the improved connectivity in the SMA pathway indicates the decrease in COP sway path length immediately after 15 min a-tDCS within the anticipatory postural adjustment processing network [30]. TDCS entails that sending weak direct currents to deep brain areas can drive neuromodulation. A systematic review and meta-analysis consistently demonstrated that the combination of a-tDCS over motor-related areas and repetitive gait training could improve gait rehabilitation in individuals with stroke [46]. Given the prominent role of the M1 leg area in executing lower extremity function, this stimulus area is a potential target for improving sensorimotor control scenarios in adults. In this regard, a-tDCS can significantly facilitate learning capabilities by evaluating task performance and kinematic variables in healthy young participants [25]. The same experimental protocol was applied to the elderly, but no positive effects of a-tDCS were found; thus, the authors hypothesised that inter-individual differences may be an unfavourable factor for this result [26]. Two cross-studies were performed to assess the corticospinal excitability and postural sway of a-tDCS applied over the M1 or cerebellum and to comprehensively understand the effect of a-tDCS on lower extremity sensorimotor control [14,18]. These studies suggest that apart from the different stimulation targets, age group, postural measure, and visual condition (eyes open or closed) can affect the ergogenic effects of a-tDCS. Furthermore, the cerebellum plays an important role in postural control. The most typical symptoms of a cerebellar lesion are decreased balance, abnormal gait, and increased risk of falling (i.e., ataxia) [47]. Therefore, the effect of cerebellar a-tDCS on postural steadiness has received widespread attention in the literature. The rationale behind using cerebellar a-tDCS as a tool in the context is that the increased activity of the cerebellum related to motor function could boost adults’ lower extremity sensorimotor control. From this point of view, a number of studies confirmed the positive effects of 20 min cerebellar a-tDCS on postural adaptation in young and older adults using the standing dynamic platform assessment system [11,32]. Contrary to these findings, another study showed that 10 min cerebellar a-tDCS with high current density (2.8 mA) had no significant effect on improving the acquisition of motor skills in young participants [2]. The authors attributed the loss of cerebellar a-tDCS effectiveness to the small sample size, inappropriate electrode position, and size. Despite the many factors that affect the effectiveness of a-tDCS on lower extremity sensorimotor control, the duration of stimulation found in the above-mentioned studies is a pivotal factor that cannot be ignored.

Investigations specific to lower extremity sensorimotor control include studies not only on gait speed indicators but also time-on-task required to perform the postural tests. Based on previous reports, time-on-task is defined as the time from the appearance of a stimulus to the completion of the response. It reflects the coordination and rapid response ability of the human nerve and musculoskeletal system, which ensures humans perform the basic daily activities. In general, a complete time-on-task cycle needs the individual to undergo stimulus identification, then select the appropriate response, and finally finish the instruction. However, ongoing studies have shown that the SMA plays an important role in movement preparation, particularly in the case of complex tasks following visual cues [48]. In addition, existing evidence suggests that a-tDCS over the SMA can significantly reduce time-on-task in dynamic balance tests, which require a more complex planning process [49]. Given the close position between the SMA and leg M1, another study on M1 a-tDCS improving ankle time-on-task in young adults hypothesised that the ergogenic effect was partly attributed to the effect of the SMA [22]. In this context, Saruco et al. [33] found that combining motor imagery practice with a-tDCS applied over the M1 can facilitate short-term motor learning by enhancing the cortical excitability of a postural task required to reach targets located forward. In the assessment of leap task time, Lee et al. [28] also pointed out that a-tDCS over the M1 can effectively improve balance performance and shorten response time in healthy young adults. However, in another study, no significant improvement was reported [23]. The TUG test is widely utilised for evaluating the mobility of dynamic posture control. Applying the stimulation of the M1, a-tDCS has been affirmed in enhancing time-on-task using the TUG test [19,21].

In the gait speed test of single and dual tasks, small sample size is still a key factor affecting the effect of a-tDCS. This greatly affects the selection of stimulation targets, stimulation duration, and intensity in the experimental protocol, and even causes inconsistencies in results. In addition, a-tDCS generally showed a positive effect on time-on-task regardless of age.

### 3.3. Limitations

This study has several limitations: (a) the searched database is limited; (b) all participants included were healthy adults; (c) the study only reviewed the effects immediately after the a-tDCS session and did not examine the longer-term follow-up effects of tDCS; and (d) the effects of cathode tDCS are not discussed in this study.

## 4. Conclusions

The ergogenic effect was observed in dual-task conditions, e.g., stimulating the dlPFC using a-tDCS had an evident development in standing task performance amongst the elderly. Meanwhile, significant enhancements in gait speed and time-on-task were observed when comparing a-tDCS with sham stimulation. In particular, a-tDCS had an effective reduction in time-on-task for the young and older population during different tests.

## Figures and Tables

**Table 1:** Characteristics of the included studies.

Reference	Trial Design	Number, Gender, and Interval Time	Age (Years)	Session and Electrode Size (cm^2^) (+/−)	Anode/Cathode Areas	Protocol
Baharlouei et al., 2020 [9] *	Double-blind, cross	(16) A-tDCS, (16) sham, (16 M/16 F), =7 days	67.59 ± 6.29	One; 27/36	M1/dlPFC; CA/right shoulder	To complete balance assessment under the single and dual-tasks condition
Craig et al., 2017 [14] #	Double-blind, cross	A-tDCS = sham = 32, (16) young (6 M/10 F) and (16) older (4 M/12 F), 3–7 days	Young (20.81 ± 2.07) Older (72.44 ± 4.03)	One; 50/25	M1/inion; CA/right BM	To complete a postural control task
Devanathan et al., 2016 [22] #	Single-blind, cross	A-tDCS = sham = 14, (6 M/8 F), 7–9 days	20–32	One; 12.5/35	M1/SA	To investigate the lower-limb simple reaction time and choice reaction time and to complete symbol digit modality test
Dutta et al., 2014 [23] #	Single-blind, cross	A-tDCS = sham = 5 (M), =7 days	22–33	One; 9/35	M1 right leg area/left OBF	To complete low-cost point-of-care testing of standing posture
Ehsani et al., 2017 [11] *#	Double-blind, parallel	(14) A-tDCS (6 M/8 F), (15) sham (7 M/8)	A-tDCS (66.08 ± 6.33), sham (65.5 ± 6.14)	One; 25	CA/right arm	To the assessment of balance and postural stability during standing on static and dynamic platforms
Hafez et al., 2018 [18] *#	Double-blind, cross	(11) Cerebellar a-tDCS (5 M/6 F), (12) M1 a-tDCS (6 M/6 F), (12) sham (7 M/5 F)	Cerebellar a-tDCS (66.91 ± 4.39), M1 a-tDCS (64.17 ± 3.48), sham (67.17 ± 4.91)	3 per week; 35	Left M1/right SA; bilateral CA/right BM	To assess the effect of the postural training on balance and postural stability
Inukai et al., 2016 [24] *	Single-blind, cross	A-tDCS = sham = 16 (M), >3 days	21.0 ± 2.9	One; 35	Inion/PC	To complete the standing posture control
Kaminski et al., 2016 [25] #	Single-blind, parallel	(12) A-tDCS, (12) sham, (12 M/12 F)	26.08 ± 3.19	One; 25/50	Bilateral M1 leg area/right PC	To complete a complex whole-body dynamic balancing task
Kaminski et al., 2017 [26] #	Cross-sectional, cross	(15) A-tDCS, (15) sham, (13 M/17 F)	A-tDCS (66.8 ± 5.63), sham (68.6 ± 6)	One; 25/50	M1 leg area/right OBF	To complete a dynamic balance task
Katagiri et al., 2020 [27] *	Triple-blind, cross	(12) A-tDCS (6 M/6 F), (12) sham (6 M/6 F)	21.8 ± 1.7	One; 35	CA/SA	To complete a visuomotor accuracy-tacking task combined with postural control training
Lee et al., 2012 [28] *#	Single-blind, cross	(15) A-tDCS (5 M/10 F), (15) sham (4 M/11 F)	A-tDCS (21.8 ± 1.3), sham (21.4 ± 1.5)	One; 35	M1/SA	To complete a dynamic posture control based on the Biodex Balance System SD
Manor et al., 2015 [29] *#	Single-blind, cross	A-tDCS = sham = 37, (12 M/25 F), =7 days	61 ± 5	One; 35	Left PC/right SA	To evaluate the postural control in single-task walking and dual-task walking
Nomura et al., 2018 [30] *#	Double-blind, cross	A-tDCS = sham = 12 (4 M/8 F), ≥14 days	72.3 ± 5.3	One; 9/35	Left SMA/right OBF	To complete rapid shoulder flexion task with self-paced 10 times on a force plate
Pineau et al., 2020 [31] *	Double-blind, cross	(12) A-tDCS (9 M/3 F), (12) sham (9 M/3 F)	21.3 ± 1.2	One; 25	Left dlPFC/right OBF	Standing on a force platform and performing a simple and dual-task with eyes open and closed
Poortvliet et al., 2018 [32] *	Double-blind, cross	(14) A-tDCS (5 M/9), (14) sham (7 M/7 F)	A-tDCS (25.64 ± 3.82), sham (25.14 ± 4.44)	One; 35/100	CA/PC	To complete a postural control on a force platform
Rostami et al., 2020 [19] #	Double-blind, cross	(16) A-tDCS (8 M/8 F), (16) sham (8 M/8 F)	60–91	5 consecutive days; 55.25	Left M1/right SA	(a) To perform Timed Up and Go Test, (b) to perform Modified Figure of Eight Walk Test, (c) to perform 30 s Chair Stand Test
Saruco et al., 2016 [33] *#	Double-blind, cross	A-tDCS = sham = 14 (8 M/6 F), =7 days	25.78 ± 3.76	One; 25/35	Bilateral M1/PC	To complete a postural control task
Schneider et at., 2021 [34] *#	Double-blind, cross	A-tDCS = sham = 25 (5 M/20 F), ≥3 days	73.9 ± 5.2	One; π	Left dlPFC and M1/FC1, CP1, AF4, FC5	To assess the dual-task walking costs in older population
Steiner et al., 2016 [2] #	Double-blind, cross	(10) A-tDCS (5 M/5 F), (10) sham (5 M/5 F)	23.7 ± 2.4	One; 35/25	CA/bilateral BM	To perform a postural control task
Takeuchi et al., 2018 [35] *	Double-blind, cross	A-tDCS = sham = 20, (9 M/11 F), ≥7 days	21.5 ± 1.1	One; 25	TC/Cz	Standing on the middle of the Wii Fit Balance Board to evaluate the postural stability
Xiao et al., 2020 [20] *	Double-blind, cross	A-tDCS = sham = 14 (M), =7 days	22.8 ± 1.2	One; 1	M1/C3, C4, Fz, Pz	To complete the assessment of passive ankle kinaesthesia, metatarsophalangeal joint flexor strength, toe flexor strength, and static balance ability
Yi et al., 2021 [36] *#	Double-blind, cross	(31) A-tDCS (10 M/21 F), (26) sham (9 M/17 F)	A-tDCS (78.13 ± 4.76), sham (78.77 ± 4.80)	One; 24	Cz	To complete a 10 m walk, static and dynamic balance tests
Zhou et al., 2014 [37] *#	Double-blind, cross	A-tDCS = sham = 20, (10 M/10 F), =7 days	22 ± 2	One; 35	Left dlPFC/right SA	To complete gait assessments on 50 m indoor walkway and the postural control on a stational force platform with serial-subtraction cognitive task
Zhou et al., 2015 [38] *	Double-blind, cross	A-tDCS = sham = 20 (11 M/9 F), =7 days	63 ± 3.6	One; 35	Left PC/right SA	Standing postural control on the stationary force platform to complete single- and dual-task
Zhou et al., 2018 [21] *#	Double-blind, cross	A-tDCS = sham = 20, =7 days	61 ± 4	One; 35	Left M1/right SA	To complete the Timed Up and Go Test and assess the standing vibratory threshold of each foot
Zhou et al., 2021 [39] *#	Double-blind, cross	A-tDCS = sham = 57 (14 M/43 F), =3 days	75 ± 5	One; 3.14	Left dlPFC and (or) SM1/FC1, CP1, AF4, FC5	To complete walk and stand with and without concurrent tasks

Note: *, the study included static test; #, the study included dynamic test. Abbreviations: M/F = male/female; dlPFC = dorsolateral prefrontal cortex; OBF = orbitofrontal cortex; SMA = supplementary motor area; TC = temporal cortex; M1 = primary motor cortex; CA = cerebellum area; PC = prefrontal cortex; SA = supraorbital area; BM = buccinator muscle.

## Data Availability

Not applicable.
